# IL-33, an Alarmin of the IL-1 Family Involved in Allergic and Non Allergic Inflammation: Focus on the Mechanisms of Regulation of Its Activity

**DOI:** 10.3390/cells11010107

**Published:** 2021-12-30

**Authors:** Corinne Cayrol

**Affiliations:** Institut de Pharmacologie et Biologie Structurale, IPBS, Université de Toulouse, CNRS, UPS, 31077 Toulouse, France; Corinne.Cayrol@ipbs.fr; Tel.: +33-5-61-17-59-53; Fax: +33-5-61-17-59-94

**Keywords:** IL-33, alarmin, protease, allergy, type-2 immunity, type-1 immunity, inflammation, asthma

## Abstract

Interleukin-33 (IL-33) is a member of the interleukin-1 (IL-1) family that is expressed in the nuclei of endothelial and epithelial cells of barrier tissues, among others. It functions as an alarm signal that is released upon tissue or cellular injury. IL-33 plays a central role in the initiation and amplification of type 2 innate immune responses and allergic inflammation by activating various target cells expressing its ST2 receptor, including mast cells and type 2 innate lymphoid cells. Depending on the tissue environment, IL-33 plays a wide variety of roles in parasitic and viral host defense, tissue repair and homeostasis. IL-33 has evolved a variety of sophisticated regulatory mechanisms to control its activity, including nuclear sequestration and proteolytic processing. It is involved in many diseases, including allergic, inflammatory and infectious diseases, and is a promising therapeutic target for the treatment of severe asthma. In this review, I will summarize the literature around this fascinating pleiotropic cytokine. In the first part, I will describe the basics of IL-33, from the discovery of interleukin-33 to its function, including its expression, release and signaling pathway. The second part will be devoted to the regulation of IL-33 protein leading to its activation or inactivation.

## 1. The IL-33/ST2L Axis: From Discovery to Function

### 1.1. Discovery and Structure of Interleukin-33 (IL-33)

The dog IL33 cDNA (called DVS27) was initially identified as a differentially expressed cDNA in canine vasospastic cerebral arteries after subarachnoid hemorrhage [1]. A few years later, the protein encoding human interleukin-33 (called NF-HEV for nuclear factor of high endothelial venule) was first characterized as a 270 amino acid nuclear factor expressed in human HEV vascular endothelial cells [2], with no sequence homology to any known protein. The human and murine *IL33* genes have been characterized: the human *IL33* gene is located on chromosome 9p24.1, and contains one non-coding exon (exon 1) and seven coding exons (exons 2–8) while the murine *IL33* gene is located in the syntenic region of chromosome 19qC1 [2]. Subsequently, IL-33 (IL-1F11) was identified by a computational approach [3] as a 30 kDa (270 aa) protein structurally related to IL-1 and fibroblast growth factor (FGF). Indeed, the C-terminal part (amino acids (aa) 112 to 270) of IL-33 has a three-dimensional structure of 12 beta-strands organized in a beta-trefoil fold, characteristic of IL-1/FGF family members [4]. IL-33 is an extracellular ligand for the orphan IL-1 receptor, IL-1RL1b (ST2), and has cytokine activity [3] making IL-33 a member of the IL-1 family. IL-33 is found only in mammals, whereas its ST2 receptor is found in birds, fish and reptiles, suggesting that there may be alternative ligands in other clades. Furthermore, IL-33 does not share a common ancestor with other IL-1 family members, making IL-33 an atypical member of the IL-1 cytokine superfamily [5,6]. Its amino-terminal portion (aa 1–65) is necessary and sufficient to bring IL-33 into the nucleus and allow its binding to chromatin [7]. It includes a chromatin-binding motif (CBM, aa 40–58) whose peptide sequence MXLRSG is essential to interact with the acidic pocket formed by the histone H2A-H2B dimer at the nucleosome surface [8]. Although early studies raised the possibility that IL-33 is a transcription factor with an HLH domain [2,7], later studies have clearly shown that IL-33 does not bind to DNA and has no transcriptional role [8,9,10,11,12,13]. Alignment of the human IL-33 protein with the mouse sequence (48% identity over 270 residues) revealed that the IL-33 protein is composed of two evolutionarily conserved regions: The N- and C-terminal domains of IL-33 show 59% and 57% identity between human and mouse, respectively, suggesting important functions of these two domains. The central domain of the protein (aa 79–111 in human) is a divergent linkage domain that serves as a cleavage and activation platform for a large number of proteases, both endogenous and exogenous, released during inflammation or infection [14,15,16].

### 1.2. Sources of IL-33 and Regulation of Expression

#### 1.2.1. Expression of IL-33

IL-33 is present, under basal conditions, in blood vessels and mucosal tissues exposed to the environment, which constitute the first line of defense against pathogens, but also in many other tissues (for a review, see [17]). In humans, IL-33 is constitutively and abundantly expressed in the nuclei of endothelial cells along the vascular tree as well as in epithelial cells and stromal cells [18] (including fibroblasts, myofibroblasts, stellate cells [19], Müller cells [20], osteoblast [21], glial, mesenchymal and smooth muscle cells [22]) in many organs such as secondary lymphoid organs, lung, stomach, liver, kidney, pancreas, small intestine, vagina, prostate, adipose tissues and brain.

The expression of IL-33 in mice resembles, overall, that found in humans, i.e., it is expressed in endothelial, epithelial and stromal cells [23,24,25,26], but probably to lower levels in mice [3]. Moreover, some species differences in the expression of IL-33 have been reported: for example, expression of murine IL-33 has been observed in the endothelium mainly during an inflammatory reaction as demonstrated in an Il-33–LacZ gene trap reporter mouse strain while it is expressed abundantly at basal level in human [18,25]. Similarly, regarding airway epithelial cells, the murine form is expressed in type II alveolar cells (ATII) at steady state [27], whereas in humans it is found in some basal bronchial epithelial cells and not in ATII cells [28]. Moreover, whereas IL-33 is constitutively expressed in mouse noninflamed skin keratinocytes, by contrast, in humans, IL-33 is weakly expressed there, in the basal state, but induced during acute inflammation [29]. Finally, while there is increasing evidence that IL-33 is abundantly expressed in the brain, notably in oligodendrocytes, astrocytes, some adult hippocampal neurons and spinal cord of mice [3,12,25,30,31,32] in adult or during embryonic and postnatal development [33], no study reports such an abundant expression in the human brain except in vascular capillaries [34] and astrocytes [35], suggesting an important difference in expression between these two species. Similarly, IL-33 expression has been reported in mesothelial cells from mice [36,37] which form a protective cobbled monolayer against physical damage, surrounding organs in the peritoneal, pleural and pericardial cavities, but, to my knowledge, there are no data yet on this localization in humans. 

Although numerous studies have indicated that IL-33 is not present in hematopoietic cells [22,38], it has also been reported that IL-33 can be expressed, under certain circumstances, by several hematopoietic cell lineages, including monocytes, macrophages, T-reg cells, dendritic cells or pre-pro-B cells in bone marrow. [3,39,40,41,42,43,44,45]. Since macrophages or dendritic cells have the ability to phagocytose cells, it is not excluded that the IL-33 expression observed in these cells is due to phagocytosis of cells that express IL-33. Furthermore, studies analyzing mRNA expression levels (such as single cell RNA sequencing; see for example, Atlas of human blood dendritic cells and monocytes, https://singlecell.broadinstitute.org/single_cell/study/SCP43/atlas-of-human-blood-dendritic-cells-and-monocytes, accessed on 21 December 2021) have clearly shown that IL-33 is either absent in immune cells or expressed at much lower levels than in non-hematopoietic cells and/or in very few cells, like in bone marrow pre-pro-B cells [3,43,46] strongly suggesting that the essential roles of IL-33 are carried by structural cells in these studies. In particular, Nakae’s group has shown that IL-33 produced by immune cells derived from bone marrow stem cells is not crucial for development of HDM-induced allergic rhinitis [47]. The question of the relative importance of IL-33 released by immune cells versus structural cells is important and remains open in some circumstances. On the other hand, Polumuri et al. described up to 40-fold induction of IL-33 mRNA by TLR agonists in macrophages, but even so, IL33 mRNA expression in these cells remains low or very low when compared to a reference gene and to other IL-33 expressing cells [46]. Showing regulation of mRNA expression by fold induction alone can be misleading and it is really informative to compare expression of an interest gene to a reference gene such as GAPDH in different cell types and conditions.

#### 1.2.2. Regulation of IL-33 Expression

Although IL-33 is constitutively expressed, its expression can also be regulated in various physiological and pathophysiological situations. Increased expression of mouse and/or human IL-33 has been observed during inflammation such as asthma [22,48,49] chronic obstructive pulmonary disease (COPD) [28], ulcerative colitis [50,51], fibrosis [52] or after parasite infestation or virus infection, such as influenza [53,54], RSV [55,56] as well as after allergen exposure or in allergic patients [57,58,59,60], after a hypo-osmotic stress [61] or exposure to cigarette smoke [53]. In addition, in these pathological conditions, other cell types/organs may express IL-33, for example keratinocytes in the skin [62] or myofibroblasts in the liver, pancreas [19] or kidneys [63].

A growing number of studies have identified some molecular mechanisms and factors involved in the regulation of IL-33 at the mRNA and/or protein level. For example, interestingly, several recent studies in mice have shown increased IL-33 production in ATII cells shortly after birth and during the alveolar phase of lung development, when the lung is remodeling [64]. It is likely that this increase in expression is due to mechanical stretching of the cells during early respirations and during lung remodeling and/or oxidative stress [65]. This wave of IL-33 expression causes a transient proliferation and activation of type 2 innate lymphoid cells (ILC2) in the neonatal lung associated with accumulation of type 2 immune cells such as eosinophil [66], M2 macrophages [64], basophil and mast cells [38]. 

Recently, a “humanized” transgenic mouse model has shown that a 5-kb non-coding regulatory element upstream of the IL33 gene, which loops with the IL33 promoter, controls the specific expression of human IL33, e.g., in lymph node HEV endothelial cells or basal lung epithelial cells [67] that do not express the endogenous mouse protein. Analysis of the *IL33* promoter in vitro, has shown that IL-33 expression can be induced by a number of factors involved in inflammation, such as TNFα (tumor necrosis factor α), IFNγ (interferon γ) or IL-1β in both human and mouse tissue and cells [21,22,51,62,68,69,70,71,72]. Moreover, IL-33 is a direct target of Notch signaling, consistent with the presence of conserved RBP-Jκ binding sites in the *IL33* gene [73]. Furthermore, several studies have shown that *IL33* expression can be modulated by miRNAs. In particular, inhibition of miR-200b and miR200c increases IL-33 expression levels in an in vitro model of lung epithelial cells, while intranasal administration of these miRNAs to mice in an in vivo model of allergic inflammation results in decreased IL-33 expression levels and resolution of airway inflammation [74]. In another study, lack of miR-155 alters ST2/IL-33R expression on ILC2s and impairs lung IL-33 production in response to acute or chronic allergen challenge [75]. Finally, Yamazumi et al. have shown that an RNA binding protein Mex-3B binds to the 3’ UTR of mouse *IL33* and post-transcriptionally activates its expression by inhibiting miR487b-3p that directly suppresses IL-33 expression itself. Moreover, mice deleted from Mex3b exhibit reduced expression of IL-33 compared to wild-type mice and develop less airway inflammation [76].

### 1.3. Release of IL-33

IL-33, like most IL-1 family members, lacks an N-terminal signal peptide and therefore bypasses the classical secretory pathway that uses the endoplasmic reticulum and Golgi apparatus. Unlike other members of the IL-1 family, such as IL-1 or IL-18, IL-33 does not require cleavage by caspase-1 to be released in active form into the extracellular space [77,78,79,80]. It is likely to be rapidly released passively from damaged necrotic cells after tissue or cellular stress [78], during viral infection [53,81] parasitic infestation [27,35,82], sterile or allergic inflammation [16,57,78,83] or after traumatic injury e.g., during ischemia reperfusion injury [84,85]. Indeed, after only 5 min of endothelial cell challenge with *Alternaria alternata* extracts, IL-33 is released in its full-length form into the extracellular space [14]. Furthermore, 15–30 min was sufficient for the release of murine IL-33 after exposure to *Alternaria alternata* extract or PLA2 allergens in vivo [14,86]. However, the precise molecular mechanism of release has not been elucidated, in particular, we do not know whether cell death is absolutely required for release and what type of death allows IL-33 release and in what form is IL-33 released. Nevertheless, at present, it is not excluded that active secretion mechanisms allow the release of IL-33 into the extracellular environment after some particular cellular stress [60]; indeed, it has been proposed that various stimuli, including ATP [87], uric acid, oxidative stress [88,89] or PAR (protease-activated receptor) protein activation, lead to IL-33 release, involving in particular an increase in intracellular calcium and/or P2 purinergic receptor (for a review, see [90]), although the precise mechanism of release remains elusive. Interestingly, recent papers suggested continuously release of low amount of IL-33 to sustain target cells at steady state in different organs, such as T-reg or ILC2 in the lung or intestine, without triggering a large-scale immune response [91,92]. In addition, using a model of conditional transgenic mice, Kita et al. demonstrated that a transient IL-33 overexpression in lung epithelial cells leads to spontaneous ST2-associated lung pathology in neonatal mice but not in adult mice, suggesting that IL-33 protein is more readily released extracellularly in developing lungs [93]. This spontaneous release could be due, in adults, to epithelial cell turnover and/or organ remodeling during early life. Indeed, the epithelium of the gastrointestinal tract has one of the highest rates of cell renewal in the human body, with a life span of 3 to 5 days [94].

### 1.4. IL-33 Membrane Receptor, ST2L, and Induced Signaling Pathways in Target Cells 

Once secreted, IL-33 binds to its specific membrane receptor, ST2L or ST2 (for serum stimulation 2; or IL1RL1b), a member of the toll-like/interleukin (IL)-1-receptor-like superfamily, that was originally identified as a gene induced by serum in mouse fibroblasts [95]. This gene is part of the IL-1 receptor gene cluster located on chromosome 2q13 in humans, and chromosome 1 in mice. The ST2L receptor is expressed on a wide range of cellular targets, notably on immune cells and its expression can be constitutive or environmentally induced [96]. It can be regulated by degradation, via binding to FBXL19, an E3 ubiquitin ligase, that induces its polyubiquitination and subsequent degradation by the proteasome [97]. Furthermore, HpBARI, a protein secreted by the parasite *H. polygyrus*, is able to bind and block ST2L, inhibiting IL-33 responses in a murine model of asthma [98]. The primary targets of IL-33 are tissue-resident innate immune cells that constitutively express the ST2L receptor, such as innate lymphoid cells type 2 (ILC2), mast cells, basophils, macrophages and regulatory T cells (Tregs) [17,90,96,99], which are able to respond very rapidly to IL-33 released into the tissue. Other targets may respond to IL-33, after induction of receptor expression on new target cells. Once IL-33 is bound to the ST2L receptor, a new binding interface is created by the dimeric complex that recruits the IL1RAcP (IL-1 receptor accessory protein) co-receptor [100,101]. Dimerization of the TIR domains of the ST2 and IL1RAcP receptors results in MyD88 binding that leads to activation of TRAF6, thereby activating the downstream NFκB and MAPK pathways. Since several members of the IL-1 family (IL-1α, IL-1β and IL-36) use the same IL1RAcP co-receptor, differential expression of their specific ST2L, IL-1R1 and IL-1RL2 receptors on target cells is, therefore, essential to explain the unique biological effects of each cytokine.

In addition to ST2L expression on target cells, cooperation or even synergy between IL-33 and other cytokines or immune mediators may also play a key role in modulating IL-33 function. For example, it has been recently shown that TSLP, an epithelial derived cytokine involved in asthma, that activates the JAK/STAT and the PI-3 kinase pathways, and IL-33 synergistically promoted group 2 innate lymphoid cell (ILC2) activation by reciprocally increasing their expression and the expression of their receptors on pulmonary ILC2 in vivo and in vitro, following *Alternaria alternata* challenge to enhance innate type-2 airway inflammation [102]. Moreover, numerous studies have shown synergy between lipid mediators such as leukotrienes, which signal through nuclear factor of activated T-cell (NFAT) and IL-33 that activates AP-1 and NFkB pathways on ILC2s responses and type 2 inflammation, in various models, such as *Nippostronglyus brasiliensis* parasite infection model or during allergen challenge with *Alternaria alternata* [103,104,105,106]. Finally, it has been demonstrated that, in a context of viral infection or chronic inflammation, IL-33 synergizes with IL-12 to promote IFNγ production and CD8+ T-cell effector function [107]; (see below; paragraph Biological Function).

In addition, heterocomplexes between IL-33 receptor and other cytokine receptor families have been described and, also, participate in immune regulation. For example, the receptor tyrosine kinase c-Kit which is the receptor for stem cell factor (SCF), constitutively interacts with IL1RacP in human and murine mast cells. Upon IL-33 stimulation, ST2L binds to this hetero-receptor, leading to c-kit phosphorylation, receptor tyrosine kinase activation and cytokine release [108]. The IL-33 receptor thus, cross-activates the c-Kit receptor that is crucial for an effective IL-33–induced signaling in mast cells. Similarly, it has been shown that IL-33 and substance P, a peptide implicated in inflammatory processes, enhance TNF synthesis and secretion from cultured human mast cells, via interaction between ST2L and substance P receptor NK-1 [109]. Moreover, during helminth infection, ST2L forms an active signaling complex with the epidermal growth factor receptor (EGFR) on Th2 cells, following amphiregulin-induced phosphorylation and activation of EGFR. This allows IL-33 to activate the MAP-kinase signaling pathway and induce phosphorylation of ERK, resulting in the expression of IL-13, which contributes to nematode clearance [110]. Because ST2L is expressed on a wide range of hematopoietic cells, it is likely that it may form a shared complex with other receptors in various cell types and their function is probably underappreciated to date.

### 1.5. Biological Functions of IL-33 and Associated Diseases

IL-33 functions as an alarm signal (or alarmin), which detects and signals the presence of tissue damage to local immune cells. Constitutive expression of preformed IL-33 in the endothelium and epithelial barriers allows it to respond rapidly to damage caused by pathogens (viruses or parasites), allergens or intrinsic injury (as during ischemia). A growing number of studies show that in addition to its role in type 2 and type 1 immunity, IL-33 regulates a plethora of physiological processes, depending on the context and the microenvironment that modulates the distribution of the ST2L receptor on target cells.

#### 1.5.1. Major Role of IL-33 in Type 2 Immune Responses and Allergic Diseases

Numerous studies have shown that exogenous administration of IL-33 to mice induces expansion and activation of innate immune cells, including ILC2, mast cells and basophils that secrete type 2 cytokines (IL-4, IL-5, IL-9 and IL-13), chemokines (eotaxin) and pro-inflammatory mediators (proteases, histamine, eicosanoids, IL-6, etc.) [17,91]. Mice treated intranasally with recombinant IL-33 produce mucus, develop eosinophilia, goblet cell hyperplasia and smooth muscle contraction leading to airway hyperresponsiveness. These responses are also observed in Rag2^−/−^ mice deficient in B/T lymphocytes, suggesting that innate cells are major targets of IL-33. Other innate immune cells (eosinophils, alveolar macrophages of M2 phenotype, dendritic cells) iNKT (invariant natural killer T) or adaptive cells (CD4^+^ T lymphocytes), recruited to the inflammatory site and expressing the ST2L receptor in a constitutive or inducible way, are also activated and/or polarized by IL-33 and participate in the inflammatory process. Thus, IL-33 plays a major role in the initiation and development of type 2 immune responses, which are essential during host infection by helminths for example [27,111,112], but which, on the other hand, contribute to the development of various allergic diseases, such as asthma or atopic dermatitis. The precise role of IL-33 in these pathologies and its mechanism of action have been widely described in various reviews [90,91,96,113,114,115,116,117]. Importantly, IL-33 plays a major role in human asthma, since the *IL33* and *IL1RL1* genes have been identified, on a recurrent basis, as susceptibility genes for asthma in very large genetic studies [118,119] (for reviews, see [17,120,121]). In addition, genetic studies in humans have demonstrated that the mutation of one allele, that results in loss of function of IL-33, reduces the number of eosinophils in the blood and protects against asthma [122]. Furthermore, after administration of itepekimab, a monoclonal antibody directed against IL-33, to patients suffering from moderate to severe asthma in phase 1 and 2 clinical trials, a decrease in blood eosinophils and an improvement in lung function were observed, suggesting that IL-33 may be a promising target for the treatment of asthma [123,124].

#### 1.5.2. Role of IL-33 in Type 1 Immune Responses

IL-33 can promote type 1 immune responses, especially in the context of chronic inflammation or infection. It had been previously demonstrated that both iNKT and human NK cells treated with a combination of IL-33 and IL-12 ex vivo produce high levels of IFN-γ [125]. In the context of COPD associated with cigarette smoke, IL-33 leads to the secretion of pro-inflammatory cytokines by macrophages and NK cells, such as TNF-α, IL-12 and IFN-γ. From a mechanistic point of view, cigarette smoke both increases IL-33 expression and, at the same time, dampens ST2 expression on ILC2s while enhancing it on NK cells and macrophages [53]. Moreover, ILC2 acquired an ILC1-like phenotype by producing IFN-γ [126]. This results in an IL-33 dependent increase in type 1 pro-inflammatory responses to virus infection and exacerbation of chronic inflammatory disease [53,126].

In other contexts, IL-33 cooperates with IL-12 produced in response to LCMV/MCMV viral infection. This cooperation allows the transient increase of ST2L receptor expression on CD8^+^ cytotoxic T lymphocytes (CTLs), the expansion and effector function of activated CTLs, and the secretion of antiviral cytokines, such as IL-10 or IFN-γ, especially by TCD8^+^, iNKT and NK cells [107,127,128,129,130,131]. Furthermore, it has been shown that loss of IL-33 or ST2 results in delayed viral clearance demonstrating that interleukin-33 is required for optimal cytotoxic CD8^+^ T cell response and antiviral immunity in mice [13,128]. The IL-33/ST2 pathway also controls coxsackievirus B5-induced experimental pancreatitis by increasing IFN-γ production by NK cells, which is associated with viral clearance [132]. Similarly, IL-33 has recently been shown to synergize with IL-12, and promote resistance to the *Toxoplasma gondii* parasite by stimulating IFNγ production by ILC1 and NK cells [82].

Recent studies show that IL-33 participates in microbial clearance by recruiting neutrophils and promoting the formation of neutrophil extracellular traps (NETs) in different pathological situations [133,134]. Indeed, it was recently shown that increased NET production by IL-33 contribute to trapping *Staphylococcus aureus* and killing bacteria in vitro and in vivo [133]. Another study indicates that IL-33, released from liver sinusoidal endothelial cells, mediates NET formation during liver I/R, exacerbating inflammatory cascades and sterile inflammation [134].

#### 1.5.3. The Other Roles of IL-33 beyond Immune Functions

A growing number of recent papers describe novel roles for IL-33 that extend beyond immunity, in particular, in the repair and homeostasis of various tissues.

Although IL-33 is involved in inflammatory processes, it also contributes to the resolution of inflammation and allows the return to homeostasis by participating in tissue repair processes. Studies show, for example, that the IL-33/ST2 axis is required to restore the integrity of the airway epithelium after infection of the lungs with influenza virus or after infestation with the nematode *Nippostrongylus brasiliensis* [91,135]. Amphiregulin, a growth factor secreted by IL-33-induced ILC2, mast cells and Treg cells, binds to EGFR and is a key component of repair [136]. Other data suggest that IL-33 is also an important regulator of tissue repair in various tissues such as skin, muscle, liver, kidney and intestine [137,138], supporting the expansion and effector function of Treg cells, directly or indirectly via ILC2 activation and M2 macrophage polarization [139,140,141]. However, when this repair process is poorly controlled, it can contribute to the development of fibrosis in different organs [142]. A reparative role for IL-33 has also been proposed in a model of traumatic spinal cord injury since oligodendrocyte recruitment and repair were decreased in *IL-33^−/−^* knock-out (KO) mice. In this model, IL-33 released from mature oligodendrocytes was shown to drive chemokine production from ST2^+^ astrocytes critical for monocyte recruitment and repair after central nervous system (CNS) injury [30].

Mounting evidence indicates that IL-33 plays important role in metabolism regulation. IL-33, produced by mesenchymal cells in pancreatic islets, promotes insulin secretion in an ILC2-dependent manner, through local myeloid cell retinoic acid production. Thus, IL-33 contributes to the regulation of islet β-cell function and mass [143]. Another role of IL-33 is the maintenance of homeostasis in adipose tissue by facilitating the differentiation and maintenance of Foxp3^+^ST2^+^ Treg cells and ILC2 in visceral adipose tissue. This results in limiting obesity-associated inflammation and preserving insulin sensitivity and glucose tolerance [144].

In addition, IL-33 appears to play an important role in homeostasis in newborns as it allows for the expansion and activation of ILC2s in the lungs of newborns [38,64,66]. It is likely that activation of ILC2s by IL-33 during the neonatal period leads to “trained” ILC2s that are able to respond more effectively to various stimuli during the adult period [145]. Type 2 immune responses are preserved throughout life and, at homeostasis, induce a basal ILC2 activation state [146], maintain alveolar macrophages in an M2-like phenotype [64], and modulate the transcriptome of lung-resident basophils [147].

Similarly, it has been proposed that IL-33 may be involved in synaptic homeostasis during early CNS development [12]. During the first postnatal weeks, the CNS undergoes extensive remodeling of synapses to ensure the formation of mature neural circuits and excessive and aberrant synaptic connections must be eliminated. Recently, a study shows that IL-33, produced postnatally by astrocytes, signals microglia to promote increased synaptic engulfment suggesting a physiological role for IL-33 in controlling synapses number in CNS development [12]. Moreover, neuronal IL-33, expressed by adult hippocampal neurons in an experience dependent manner, instructs microglial engulfment of the extracellular matrix (ECM) that drives experience-dependent synapse remodeling in the hippocampus [31].

Removal of unnecessary cells or tissues, such as atretic follicles after ovulation, is important to preserve tissue integrity and functionality. IL-33 could also participate in such a mechanism by promoting the physiological elimination of atretic ovarian follicles. Indeed, impaired macrophage migration and decreased autophagy in follicular cells were observed in *IL33^−/−^* KO mice, leading to accelerated decline in ovarian function and reduced reproductive life span [148].

Thus, it appears that IL-33 may play a general role in the control of tissue homeostasis by recruiting and activating phagocytic cells that clear debris and promote recovery, both in the neonatal and adult stages.

#### 1.5.4. IL-33 and Diseases

Given the many functions of IL-33, it is not surprising that it is associated with many diseases and the subject has already been extensively covered by various reviews [90,91,96]. In addition to the allergic diseases mentioned above, IL-33 is associated with many chronic inflammatory and/or fibrotic pathologies affecting various organs, such as the intestine in the case of ulcerative colitis or the lung in the case of COPD [113,114,116]. It is also involved in infectious diseases (sepsis, infection by worms, viruses or bacteria), cardiovascular diseases (atherosclerosis), renal diseases, metabolic diseases and in tumors, having either a protective or a detrimental role in these pathologies [90,91,96,149]. The IL-33/ST2 pathway could therefore be a pharmacological target of choice for the treatment of some diseases. For example, encouraging results have recently been obtained from phase 2 clinical trials showing that the use of the anti-IL-33 mAb, itepekimab, reduces the rate of exacerbation and improves lung function in former smokers with COPD [150].

## 2. Regulation of IL-33

**Figure 1:**
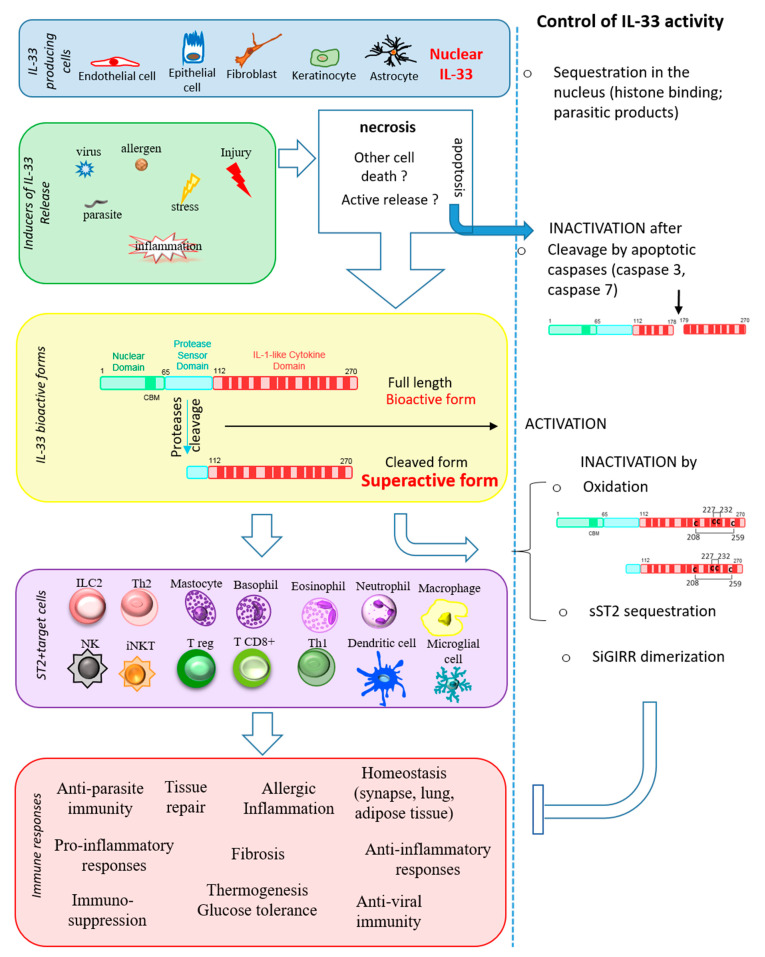
Mechanisms regulating IL-33 bioactivity. The figure shows IL-33 producing cells, inducers of IL-33 release, IL-33 bioactive forms, ST2^+^ target cells implicated in type 1 and type 2 immune responses (**left** panels) and the mechanisms regulating IL-33 activity (**right** panels).

IL-33 is stored as a full-length protein in the cell nucleus, under steady-state conditions, but upon cellular damage, it is rapidly released into the extracellular space and functions as an endogenous danger signal. The IL-33 protein must be finely regulated to increase its activity when needed or, on the contrary, to prevent its activity from becoming harmful. Several molecular mechanisms have been described that are involved in the regulation of its activity (see Figure 1).

### 2.1. IL-33 Sequestration in the Nucleus

Since IL-33 has many target cells and exerts a pro-inflammatory role, it is important that it is not present in large amounts in the extracellular medium at homeostasis. This is most likely one of the reasons why IL-33 is bound to chromatin, sequestered in the nucleus of cells, so that it does not induce an inappropriate immune response. Indeed, an elegant study using a knock-in mouse model showed that removal of the amino-terminal domain of IL-33, which includes the chromatin-binding domain, results in the loss of IL-33 from the nucleus and its extracellular release, leading to massive eosinophilic and neutrophilic inflammation on multiple organs in the animal, and death of the mice at three months of age [151]. This inflammation is dependent on the ST2 receptor showing that the observed effects are mediated by the release of IL-33 into the external environment, and thus are not due to the loss of putative function of IL-33 inside the nucleus. Similarly, a recent paper showed that expression of a form of IL-33 deleted from the first 68 residues in neurons results in local release of IL-33 sufficient to promote its function, i.e., dendritic spine formation and ST2- dependent plasticity [31], again indicating that the N-terminal domain of the protein plays a critical role in maintaining IL-33 in the nucleus. 

Several in vitro and in vivo studies using transcriptomic and proteomic approaches as well as *IL33* and *ST2* KO mouse models, have demonstrated that IL-33 located in the nucleus of endothelial or epithelial cells has no effect on gene transcription [9,10,11]. Moreover, RNA sequencing experiments performed on fibroblasts or astrocytes from *WT* and *IL33* KO mice, show that IL-33 does not induce any significant change in gene expression [12,13]. Altogether, it is likely that IL-33 does not have an active role in the nucleus, but is retained there to limit its function as an extracellular inflammatory cytokine. This localization of IL-33 in the nucleus has been preserved during evolution, probably to maintain tissue homeostasis and protect the organism from uncontrolled lethal inflammation. 

### 2.2. IL-33 Trapping by the Soluble sST2 Receptor

There are four isoforms of ST2, encoded by the *IL1RL1* gene located on chromosome 2q12 in humans and chromosome 1 in mice. The two most important isoforms, resulting from alternative splicing, include the transmembrane ST2 receptor (ST2L), which contains the extracellular, transmembrane and intracellular Toll-IL-1R (TIR) cytoplasmic domains, and a soluble, circulating truncated form of ST2 protein (sST2) that lacks the transmembrane and intracellular domains and includes a unique nine amino acid C-terminal sequence [152]. The soluble form of sST2 is abundantly expressed and secreted (in normal plasma, 12–26 ng/mL; [153]) by many cell types, including mast cells [154], epithelial cell, Th2 T cells [155,156] or fibroblasts [157], from numerous tissues such as lung, kidney, heart and small intestine [158,159]. It acts as a decoy receptor by binding to IL-33, neutralizing free IL-33 in biological fluids, thereby limiting IL-33-induced responses [155], and preventing systemic effects of the cytokine in the blood. High levels of sST2 are found in the serum of patients with inflammatory and heart diseases [155]. They are generally correlated with the severity of the disease and are considered a valuable prognostic marker in both conditions [160]. Interestingly, a counterregulatory effect of IL-33 on sST2 mRNA expression has been demonstrated: when levels of IL-33 released into the extracellular medium increase, following trauma for example, expression of the soluble sST2 receptor (mRNA and protein) also increases, probably to compensate for the severe effects of IL-33 [153,158,161,162]. 

### 2.3. Inactivation of the IL-33/ST2 Axis by the Receptor SIGIRR

In addition to IL-33, the IL-33/ST2 signaling pathway can also be regulated. An example of this regulation is provided by the negative regulator of the IL-33/ST2 pathway, SIGIRR (single immunoglobulin IL-1R-related molecule), also known as TIR8 or IL-1R8 [163,164,165]. The human SIGIRR protein is composed of a single extracellular Ig domain, a transmembrane domain, and a cytoplasmic TIR domain with an atypical tail of 95 residues. It has been proposed that SIGIRR forms a complex with ST2 upon stimulation by IL-33 and interferes with the recruitment of the IL1RAcP adaptor molecule containing the TIR domain. It, thus, inhibits the IL-33/ST2-mediated signaling pathway that leads to the production of Th2 cytokines [163]. Furthermore, the IL-33-induced Th2 response is enhanced in *SIGIRR^−/−^* knockout mice, suggesting a negative regulatory role of SIGIRR in IL-33/ST2 signaling in vivo. Among other things, an increase in IL-5 and IL-13 secretion, splenomegaly and lung inflammation are observed in these mice. Furthermore, exacerbated Th2 immune responses were observed in mice lacking *SIGIRR* in an OVA-induced asthma model, indicating that SIGIRR controls allergic inflammatory responses by suppressing IL33/ST2 signaling [163]. 

### 2.4. Inactivation of IL-33 by Oxidation

The biological activity of IL-33 is regulated by oxidation [166]. Active IL-33 is released into the extracellular environment in its reduced form and is rapidly (within 2 h) oxidized and inactivated under physiological conditions. Indeed, the oxidation of the four cysteine residues located in its C-terminal domain (Cys208, Cys227, Cys232 and Cys259) induces the formation of two disulfide bridges and a conformational change of the protein which leads to the loss of receptor binding interface resulting in its inactivation. In agreement with these results, mutation of these four cysteines prolongs IL-33 activity in the lung of humanized IL-33 mice. Both reduced and oxidized forms of IL-33 can be detected in sputum from patients with moderate to severe asthma. Similarly, an oxidized form of IL-33 has been found in bronchoalveolar lavages from mice challenged with *Alternaria alternata* extract [166]. The rapid oxidation of IL-33, thus, limits the range and duration of its action; therefore, the first hours after its release into the extracellular environment are critical for target cell activation. 

### 2.5. Activation of IL-33 by Binding to Histones

A recent study showed that IL-33 was released from necrotic cells in association with histones, particularly H2B. The authors showed that this complex had more potent activity than IL-33 alone [10]. However, the precise mechanism as to why this association increases activity has not yet been elucidated. It is likely that the binding of the N-terminal domain of IL-33 to histones prevents interference of this domain with the C-terminal portion responsible for cytokine activity. A similar mechanism, in which the nuclear basic domain might somehow interfere with the binding of the acidic cytokine domain to the ST2 receptor, has been proposed to explain why the cleaved IL33 proteins had increased activity [15]. Furthermore, we cannot exclude that histone binding may have an effect on IL-33 folding, stability or post-translational modifications that could influence its activity. 

### 2.6. IL-33 Regulation by Parasitic Products

A 26kDa protein, HpARI (H. polygyrus alarmin release inhibitor), secreted by the parasite *Heligmosomoides polygyrus,* has been shown to bind to the active reduced form of IL-33 and to genomic DNA via its N-terminal pair of complement control protein (CCP) modules (CCP1/2) in humans and mice [167]. This double binding prevents *Alternaria alternata* or freeze-thaw treatment-induced IL-33 release by keeping it inside necrotic cells. The HpARI protein is thus able to inhibit the interaction of IL-33 with ST2 and to inhibit the initiation of type 2 allergic responses induced by allergen administration or following *Nippostrongylus brasiliensis* infestation, thus increasing the parasite load. These results suggest that *H. polygyrus* can evade antiparasitic responses by scavenging IL-33. Interestingly, it has been shown that an artificial truncated form of HpARI stabilizes IL-33, on the contrary to the full-length, increasing its half-life and amplifying responses to the cytokine [168]. Future studies will be needed to identify other microorganisms (or derivatives), such as viruses, bacteria or parasites, capable of developing strategies to evade the action of the IL-33/ST2 pathway, for example by modulating IL-33 expression, stability or conformation.

### 2.7. Regulation of IL-33 by Proteases

IL-33 is a substrate for many proteases that regulate its activity, either by activating or inhibiting it.

#### 2.7.1. Activation of IL-33

Although the full-length form of IL-33 is active (at a certain dose) [77,78,79,80], proteolytic maturation plays an important role in the regulation of IL-33 activity. Indeed, it has been first shown that during inflammation, serine proteases released by neutrophils such as cathepsin G and elastase can cleave human full-length IL-33_1__–270_ and generate the mature forms IL-33_95__–270_, IL-33_99__–270_ and IL-33_109__–270_ [16], leaving an intact IL-1-like cytokine domain. Neutrophils are amongst the first cells recruited to the site of an infection or damage, and once activated, release their proteases capable of cleaving and regulating the activity of various mediators of inflammation [169]. Cellular assays have shown that these bioactive forms are produced by purified proteases and also by activated human neutrophils ex vivo [16], contrary to what may have been mentioned in some papers [170], and have ~10-fold greater biological activity than full-length IL-33. In the same study, it has been shown that murine IL-33 is also cleaved by cathepsin G and neutrophil elastase, generating an IL33_102__–266_ fragment. Indeed, both full-length and cleaved forms of IL-33 could be detected in bronchoalveolar lavage fluid in an in vivo model of acute lung injury associated with neutrophil infiltration [16]. Later, these results have been confirmed by at least two other papers, in vitro [170] and in vivo [171]. Similarly, several studies have shown that PR3 converts inactive human and murine IL-33 precursor proteins into biologically active forms [16,172] before abrogating IL-33 activities, when increasing PR3 incubation time or using excessive protease concentration [170,172]. Neutrophil-derived proteases may therefore act as physiological positive regulators of IL-33 during inflammation. 

Other studies have shown that the microenvironment exacerbates IL-33 functions during allergic inflammation. In particular, mast cells, which are present in the mucosa, play important roles in the context of allergic inflammation by secreting preformed mediators, such as chymase and tryptase proteases. These purified serine proteases cleave the recombinant full-length 33 kDa IL-33 to generate forms of IL-33 of about 19 kDa (IL-33_95__–270_, IL-33_99__–270_ and IL-33_109__–270_) which have a 10 to 30 times higher biological activity, especially on ILC2s ex vivo [15]. Moreover, supernatants from human mast cells activated with substance P or anti-IgE, that contain serine proteases, process full-length IL-33 into shorter bioactive mature forms. Murine IL-33 is also cleaved by mast cell tryptase, and a tryptase inhibitor reduced IL-33–dependent allergic airway inflammation in vivo [15]. Thus, mast cells are critical amplifiers of IL-33-mediated inflammation. It has also been proposed that IL-33 could be degraded by some mast cell proteases to limit inflammation during allergic reaction [173]. In fact, high amounts (probably not physiological) of mast cell proteases and long time incubation (>2 h) clearly leads to IL-33 degradation in vitro (personal communication). It is not known whether this actually affects IL-33 activity in vivo since IL-33 is anyway rapidly inactivated by oxidation (after 2 h in the serum) leaving enough time for IL-33 to activate target cells nearby. Moreover, the presence of endogenous inhibitors limits the proteolytic activity of these endogenous proteases. Altogether, these studies show that multiple endogenous inflammatory proteases participate in the maturation process of IL-33 by cleaving and activating it. Although the cleavage site differs from one enzyme to another, in the end, each of the proteases plays a similar role with respect to IL-33 but under different physiological conditions: indeed, it is likely that neutrophil proteases activate IL-33 in virus-induced asthma exacerbations and other inflammatory or infectious conditions, whereas mast cell proteases may be essential for IL-33 activation in allergic asthma and allergic inflammation. This same maturation process, repeated under different physiological circumstances, shows the importance of cleavages in the control of IL-33 bioactivity.

We and others have shown that the full-length form of IL-33 (IL-33FL) functions as a protease sensor that detects the proteolytic activity of environmental allergens, whether fungi, mites, cockroaches or pollens [14,86]. We were able to demonstrate both in vitro and in vivo that in the presence of allergen extracts (or purified allergen proteases), IL-33 is rapidly cleaved (<15 min) in its central “sensor” domain, releasing the IL-1 cytokine domain which leads to the production of type 2 cytokines by ILC2 and to allergic airway inflammation. Prevention of IL-33FL cleavage by the use of antibodies specifically directed against this “protease sensor” domain or by the use of protease inhibitors reduces allergic airway inflammation [14]. Interestingly, it was clearly established by a kinetic study that full size endogenous IL-33 is first released from endothelial cells into the extracellular medium after 5 min of incubation with *Alternaria alternata*. This release is independent of the proteolytic activity of the allergens and is followed by extracellular cleavage as early as 10 min after allergen exposure [14]. In vivo, cleaved forms of endogenous mouse IL-33 were the major forms detected in broncho-alveolar lavage fluids (BALFs) after a single intranasal exposure to *Alternaria alternata*, whereas uncleaved IL-33FL was the major form detected after exposure to PLA2, an allergen component that has no protease activity. These results suggest that release of IL-33 is uncoupled from its cleavage that occurs outside the cell. Furthermore, environmental allergens trigger inflammation via their proteases, cleaving and activating IL-33 directly. Since the mature forms of IL-33 (18–21 kilodaltons) are much more active than the full-length form, cleavage of IL-33FL by proteolytic activities associated with environmental allergens probably plays a critical role in the initiation of allergic inflammation by lowering the threshold for activation of IL-33-mediated responses. 

In one of these studies it was proposed that intracellular cleavage of IL-33 by calpains in vitro could lead to bioactive forms [86]. However, the authors show in the same paper that this does not occur in vivo, thus confirming that IL-33 is probably not a physiological target of calpain, and that endogenous IL-33 is not cleaved intracellularly in vivo.

**Figure 2:**
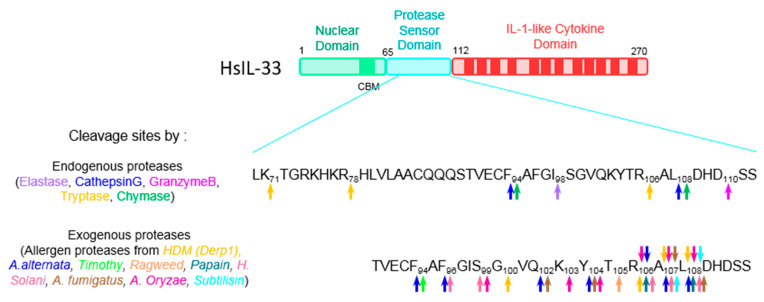
Activation of protein IL-33 by cleavage in the protease sensor domain. The figure shows the three domains of human IL-33 protein. The N-terminal nuclear domain contains a short chromatin binding motif (CBM). The central protease sensor domain is a platform of cleavage for various endogenous and exogenous proteases. The C-terminal IL-1 cytokine domain mediates binding to ST2. The cleavage sites for endogenous inflammatory proteases and exogenous allergen proteases are indicated on the human IL-33 sequence.

The data obtained with neutrophil, mast cell and allergen proteases clearly show that these “activating” proteases cleave IL-33 in the central domain from residues 72 to 110, which constitutes a true cleavage platform for “activating” proteases, whether endogenous or exogenous (see Figure 2). 

The primary sequence and cleavage sites are not conserved between species, yet the overall function of this domain is conserved, since in both humans and mice, cleavage in this domain results in IL-33 hyperactivity. One question that remains is why the N-ter domain must be eliminated for IL-33 to be fully functional? From a mechanistic point of view, it is tempting to propose that the basic N-ter domain interferes with the C-ter domain through electrostatic bond interactions, thus hindering its binding to the ST2 receptor. The crystallographic structure of full-length IL-33 could certainly answer this question in the future, although it is a real challenge.

#### 2.7.2. Inactivation of IL-33

Schmitz and colleagues originally proposed that caspase-1 cleaves human IL-33 at serine position 111 and activates it, with reference to other members of the IL-1 family, notably IL-1β [3]. However, this putative cleavage site did not correspond to a consensus caspase-1 cleavage site and is not conserved between species. A few years later, several groups demonstrated that IL-33 is not cleaved by caspase-1, and that the activity of IL-33 does not depend on caspase-1 cleavage. In contrast, the apoptotic caspases, 3 and 7, cleave human IL-33 at amino acid D178, at the D_175_GVD_178_ consensus site, generating two fragments unable to bind ST2: an amino-terminal fragment 1-178, which is still capable of binding chromatin, but has no biological activity and a C-terminal fragment 179-270, which is also inactive [77,78,79,80]. This cleavage site is located in the IL1-like C-terminal domain at an externally exposed loop between beta-sheets 4 and 5 that is evolutionarily conserved but not present in other IL-1 family members. This cleavage by caspases would therefore be specific to IL-33, and could be a mechanism to inactivate the pro-inflammatory cytokine activities of IL-33 during apoptosis, a process that does not trigger inflammation in vivo.

A very recent study argues that allergens, notably *Alternaria alternata*, trigger RIPK1-caspase-8 ripoptosome activation in epithelial cells, followed by IL-33 cleavage inside the cells by caspase-3 and -7 and extracellular release [174]. In this paper, the authors proposed that intracellular IL-33 cleavage leads to the release into the extracellular space, of an active form of IL-33_1__–175/178_, with an activity similar to that of full-length IL-33. However, there are several concerns in the way the experiments were conducted and, in the conclusions, reached. Firstly, it seems that the systematic use of a proteasome inhibitor (MG132) in the culture medium (see material and methods section of Brusilovsky et al. [174]), known to favor apoptosis [175], and to modify the ripoptosome itself [176] may have distorted the results and their subsequent interpretation in this study. How can we explain that a protease such as caspase-3 or -7 cleaves IL-33 at a motif present in an evolutionarily conserved loop that is not found in other members of the IL-1 family, only to have no additional role compared to the non-cleaved form? Secondly, several studies have shown that the C-terminal part of the protein is critical for bioactivity. For example, in a study by Bae et al., the PR3-derived forms, IL-33_1__–220_ and IL-33_1__–240_, have no activity compared to a highly active IL-33_117__–270_ form [172], suggesting that deletion of the last 50 amino acids of the IL-33 protein has a dramatic effect on its activity. Similarly, Smith et al. demonstrated that a variant form of nuclear IL-33, deleted from the last 66 residues, present in the Icelandic population, reduces blood eosinophil counts and protects from asthma. The C-terminal domain (95–204) corresponding to this form is not able to bind to the ST2 receptor, contrary to the 95–270 form, and reveals a complete loss of cytokine activity [122] suggesting, once again, that the last residues of IL-33 are absolutely necessary for IL-33 function. 

## 3. Conclusions and Perspectives

It therefore appears that the expression of the IL-33 protein must be finely regulated so that it can fully perform its alarmin function in case of danger, without becoming harmful and participating in the development of various acute or chronic pathologies. A growing body of work shows that IL-33, beyond its alarmin function, behaves as a pleiotropic cytokine, and does not act alone. Studying the cooperation of IL-33 with other regulators in homeostasis or in pathological contexts should provide a more complete and complex view of its functions. Although great progress has been made in recent years, in particular the discovery of ILC2 target cells, many aspects of IL-33 biology are not well understood, including its involvement in ageing and in the nervous system and some fundamental important biological questions remain, in particular: How is IL-33 expression regulated at the mRNA and protein level in different physiological and pathophysiological contexts? When and how is IL-33 released into the extracellular environment? What are the molecular mechanisms involved in IL-33 release? There is no doubt that the growing interest of biologists and pharmaceutical laboratories in IL-33 should allow these questions to be answered rapidly.

There are many articles describing the expression and quantification of IL-33, in tissues and biological fluids, in different physiological conditions, whether by histology, ELISA, FACS, Western blot, etc. Unfortunately, the use of tools (especially antibodies) not validated in the specific application used, can lead to erroneous conclusions, as described for another protein of interest [177]. The use of KO mice and/or siRNAs and/or multiple antibodies should be systematic in order to consider the results obtained as reliable. It should be noted that the quantification of IL-33 in human samples (tissues or biological fluids) is currently limited by the use of ELISA kits which lack sensitivity and specificity. There is therefore an urgent need to develop methods to detect full-length and cleaved forms, free and partner-bound forms, as well as reduced and oxidized forms, in order to generate reliable and usable data, especially in humans. In addition, special attention should be paid to studies that use reporter mice for expression data. Indeed, one paper has shown that depending on the reporter line, one may not have the same expression profile [178]. This may be due to the fact that the lines do not have the same genetic heritage or the same microbiota, or simply because with promoter driven reporters, it is the RNA that is analyzed and not the protein of interest; the mRNA and the protein may not be regulated the same way; finally, reporter mice that use long half-life fluorescent proteins, such as GFP, can mask the subtleties of gene regulation because fluorescent proteins may not have the same half-life or regulation as the protein of interest. Ideally, histochemical approaches that analyze protein expression should be combined with the use of reporter mice that analyze promoter activity, as previously performed in some studies [25].
